# Percolation effect of a Cu layer on a MWCNT/PP nanocomposite substrate after laser direct structuring and autocatalytic plating

**DOI:** 10.1039/c8ra04813d

**Published:** 2018-08-28

**Authors:** Mindaugas Gedvilas, Karolis Ratautas, Aldona Jagminienė, Ina Stankevičienė, Nello Li Pira, Stefano Sinopoli, Elif Kacar, Eugenijus Norkus, Gediminas Račiukaitis

**Affiliations:** Center for Physical Sciences and Technology Savanoriu Ave. 231 LT-02300 Vilnius Lithuania mgedvilas@ftmc.lt; Group Materials Labs, Centro Ricerche Fiat S.C.p.A. Strada Torino 50 10043 Orbassano (TO) Italy; BioAge Srl Via Dei Glicini 25 Lamezia Terme (CZ) 88046 Italy; Department of Physics, Faculty of Arts and Sciences, Kocaeli University Umuttepe Campus 41380 Kocaeli Turkey; Laser Technologies Research and Application Center, Kocaeli University Basiskele 41275 Kocaeli Turkey

## Abstract

Percolation behavior of a copper (Cu) layer on a multi-walled carbon nanotube/polypropylene (MWCNT/PP) nanocomposite substrate after laser-direct-structuring (LDS) and subsequent autocatalytic Cu deposition (ACD) is presented. The inverse sheet resistance showed percolation type dependence on the area fraction of Cu on MWCNT/PP measured by digital image processing of specimen photos.

## Abstract 2

Digital image processing of Cu layer on MWCNT/PP nanocomposite substrate after laser direct structuring and autocatalytic plating reveals percolation effect.

## Graphical abstract

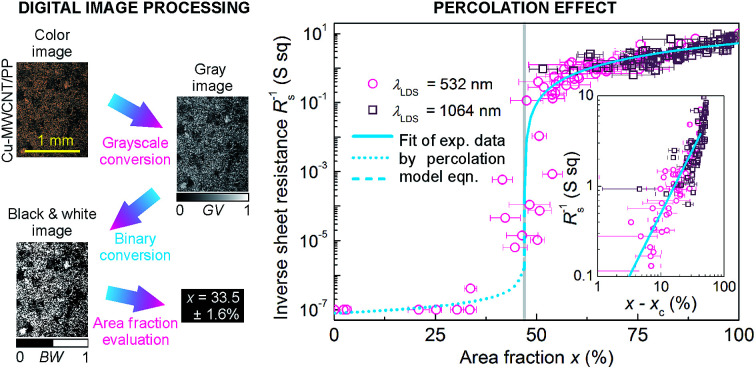

## 1. Introduction

Electronic appliances on plastic substrates are widely utilized in various fields like the automotive industry, medicine and consumer electronics.^1–4^ Fabrication of the monolithic integrated electric circuits on plastics is necessary for those application areas. Several methods are usually applied to make an electric circuit on plastic surfaces: laser-direct-imaging,^5^ laser-induced selective activation (LISA)^6–10^ and laser-direct-structuring (LDS).^11–14^

The LDS method was invented for metal deposition selectively on plastic surfaces.^15^ LDS uses precursors mixed in a plastic substrate matrix. These additives of the precursor are activated during the laser writing process, and the laser-scanned area can be selectively plated with metals. There are several commercially available precursor materials commonly used for the LDS process. However, the majority of them are expensive metal–organic fillers, usually based on palladium.^15^ The multi-walled carbon nanotube (MWCNT) has been introduced as a cheaper precursor replacement for the expensive palladium based additives in our previous work.^13^ The main reasons why MWCNT was successful precursor additive has been investigated in our research paper.^14^ By employing Raman spectroscopy and scanning electron microscopy, we have revealed that LDS does several key changes to MWCNT mixed in polypropylene (PP) substrate and what makes selective electroless copper (Cu) plating possible. The MWCNT/PP nanocomposite was melted by the laser, and MWCNT additives reoriented into the connecting structure by clustering of carbon additives and decreasing the number of defects in crystalline phase of carbon. Successive procedure to LDS is the autocatalytic Cu deposition (ACD) on the laser-patterned plastic surface with the activated precursor. By using collinear four-point probe technique, we have demonstrated that increased electrical conductivity of the laser-activated areas enabled the catalytic reaction of reducer in the electroless plating bath.^14^

The sheet resistance is the most critical electrical characteristic of the conductive layer defined by the inverse of the product of layer thickness and specific electrical conductance.^16^ However, the porous Cu layer deposited on the laser patterned plastic does not have well-defined metal/plastic boundary.^17^ Therefore, the thickness of the deposited Cu layer cannot be characterized by known techniques: atomic force microscopy, stylus profilometry, ellipsometry, interferometry, *etc.* Moreover, the specific electrical conductance of the autocatalytic deposition of Cu film might be up to 10 times lower than of bulk.^18^ Consequently, it is not a trivial task to characterize the amount and quality of the deposited Cu layer on laser-structured substrate because of hardly definable specific electrical conductance and layer thickness values.

The electric *versus* optic characteristics of the semi-transparent conductive layers were analyzed in numerous scientific works.^19–26^ However, the transmittance dependence on the thickness was investigated for ultra-thin flat films with the depth of absorption comparable to layer thicknesses. Therefore, it is not possible to apply the mentioned technique to the non-uniform Cu films on porous laser-patterned opaque plastic surfaces.

The percolation behavior of the conductive layer depending on transparency has been investigated in numerous scientific works.^27–30^ Also, percolation of the thin film after the ACD procedure has been explored.^31–33^ Moreover, the percolation behavior of sheet resistance dependence on the color-difference between sample images after LISA of precursor additive-free polymer and subsequent electroless Cu deposition procedures have been reported in our previous work.^10^ However, there is not scientific work found in literature where percolation effect has been investigated for the Cu layer on nanocomposite material with precursor additives after LDS and ACD treatment. The main idea presented in this paper is to investigate the percolation effect of a rough metal layer on the laser-patterned nanocomposite surface using digital image processing technique.

In this work, percolation effect of Cu layer on MWCNT/PP nanocomposite substrate after LDS and ACD is presented. MWCNT/PP substrate was patterned by the LDS procedure and then plated with a layer of Cu by the ACD method. The area fraction of Cu on MWCNT/PP was measured by digital image processing of specimen photos. The inverse sheet resistance of Cu layer had a power-law dependence on the difference of area fraction and percolation threshold. The percolation model of the electrical conductivity of metal–plastic compound has been applied for clarification of experimental data.

## 2. Results and discussion

**Fig. 1:**
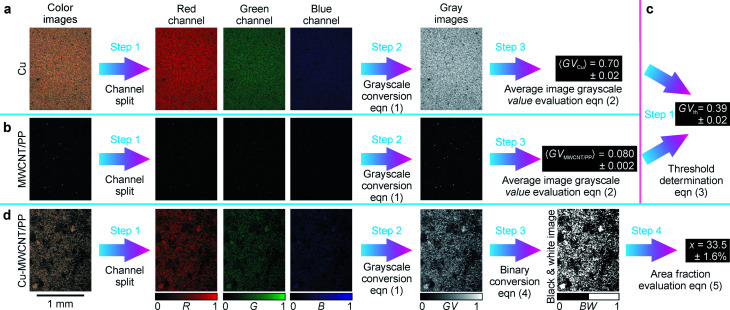
Digital image processing procedure designed for area fraction calculations. (a) Average value calculation of Cu grayscale image: step 1 – color image split to red (R), green (G) and blue (B) channels; step 2 – color-to-grayscale conversion by eqn (1); step 3 – average grayscale value of 〈GV_Cu_〉 = 0.70 ± 0.02 evaluation by eqn (2). (b) Average value calculation of MWCNT/PP grayscale image: step 1 – color image split to R, G and B channels; step 2 – color-to-grayscale conversion by eqn (1); step 3 – average grayscale value of 〈GV_MWCNT/PP_〉 = 0.080 ± 0.002 evaluation by eqn (2). (c) Image value threshold determination: step 1 - threshold of GV_th_ = 0.39 ± 0.02 calculation by eqn (3). (d) Example of area fraction calculations of Cu layer on MWCNT/PP substrate: step 1 – color image split to R, G and B channels; step 2 – color-to-grayscale conversion using eqn (1); step 3 – conversion of grayscale image to black-and-white binary image using eqn (4); step 4 – calculation of the area fraction of *x* = 33.5 ± 1.6% by eqn (5).

The MWCNT/PP nanocomposite of 2.5 wt% was selected as a substrate for the LDS and ACD procedures. This material was introduced as a successful replacement for the commonly used palladium-based expensive precursor additives in the polymer matrix in our previous works.^13,14^ The details of LDS and ACD procedures used for Cu layer deposition on MWCNT/PP nanocomposite are given in ref. 14. The sample pictures at different amounts of Cu deposited on MWCNT/PP were taken by using an optical microscope and charge-coupled device camera with the experimental details given in ref. 10. The digital image processing procedure used for area fraction calculations of Cu layer on MWCNT/PP substrate is presented in Fig. 1. In the first step, the color image was split to red (R), green (G) and blue (B) channels (Fig. 1a, b and d). In the second step, the image was transformed to the grayscale mode (Fig. 1a, b and d) by calculating grayscale value GV using the formula:^34^

1 GV = max(R, G, B),

where R, G and B are the red, green and blue components, respectively. The five color-to-grayscale conversion methods have been tested in our work: intensity, luminance, luma, luster and value.^34^ The value method has been chosen because of the highest achieved contrast between grayscale images of Cu and MWCNT/PP. In the third step, the average values of the grayscale Cu image (Fig. 1a) and grayscale MWCNT/PP image (Fig. 1b) were calculated by using the averaging equation:2
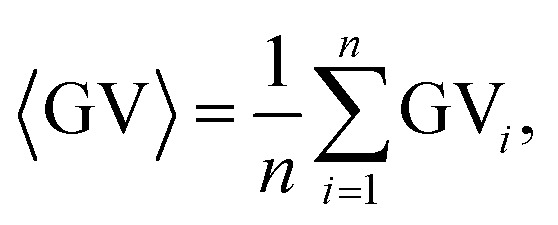
where the average is denoted by angle brackets, *n* is a total number of picture pixels, *i* is the pixel index, GV_*i*_ is the grayscale value of each pixel in the picture. The standard deviation as a computational error in the average grayscale value 〈GV〉 evaluation were taken from five sections of the microscope images. The image with the lowest measured sheet resistance of 0.1 Ω sq^−1^ was chosen for average grayscale value evaluation of Cu deposition of 〈GV_Cu_〉 = 0.70 ± 0.02 by using eqn (1) and (2) (Fig. 1a). The image with the largest sheet resistance of 10^7^ Ω sq^−1^ was chosen for average grayscale value evaluation of MWCNT/PP nanocomposite of 〈GV_MWCNT/PP_〉 = 0.080 ± 0.002 by using eqn (1) and (2) (Fig. 1b). The threshold was selected as a mean of the average values of Cu and MWCNT/PP grayscale images (Fig. 1c):3
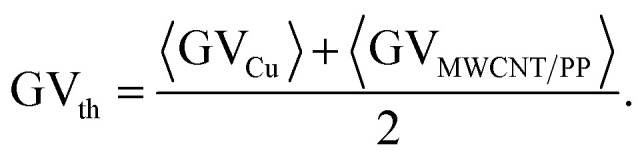


The threshold value calculated by eqn (3) was GV_th_ = 0.39 ± 0.02 (Fig. 1c). In the third step of the area fraction calculations of Cu layer on MWCNT/PP substrate, the grayscale pictures were converted to the black-and-white binary mode by using specific threshold value GV_th_ (Fig. 1d).4
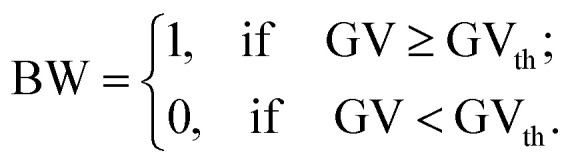


The area fraction *x* of the Cu on MWCNT/PP in percent was calculated in the fourth step by averaging equation of binary image intensity (Fig. 1d):5
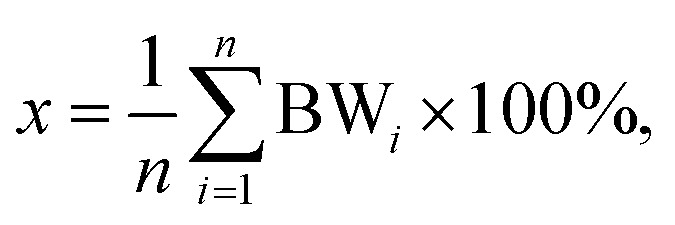
where *n* is a total number of picture pixels, *i* is pixel index, BW_*i*_ is the binary black-and-white intensity of each pixel in the picture. The computational error in the area fraction *x* evaluation was taken as the standard deviation of measurements from five sections of the microscope images. The digital image processing and area fraction characterization were performed by using a symbolic and numeric computing environment and software Maple from Maplesoft.

The collinear four-point probe technique was employed for characterization of the sheet resistance of the Cu deposition.^35,36^ The detailed experimental setup of the sheet resistance measurement used in this works is given in ref. 10 and 14.

**Fig. 2:**
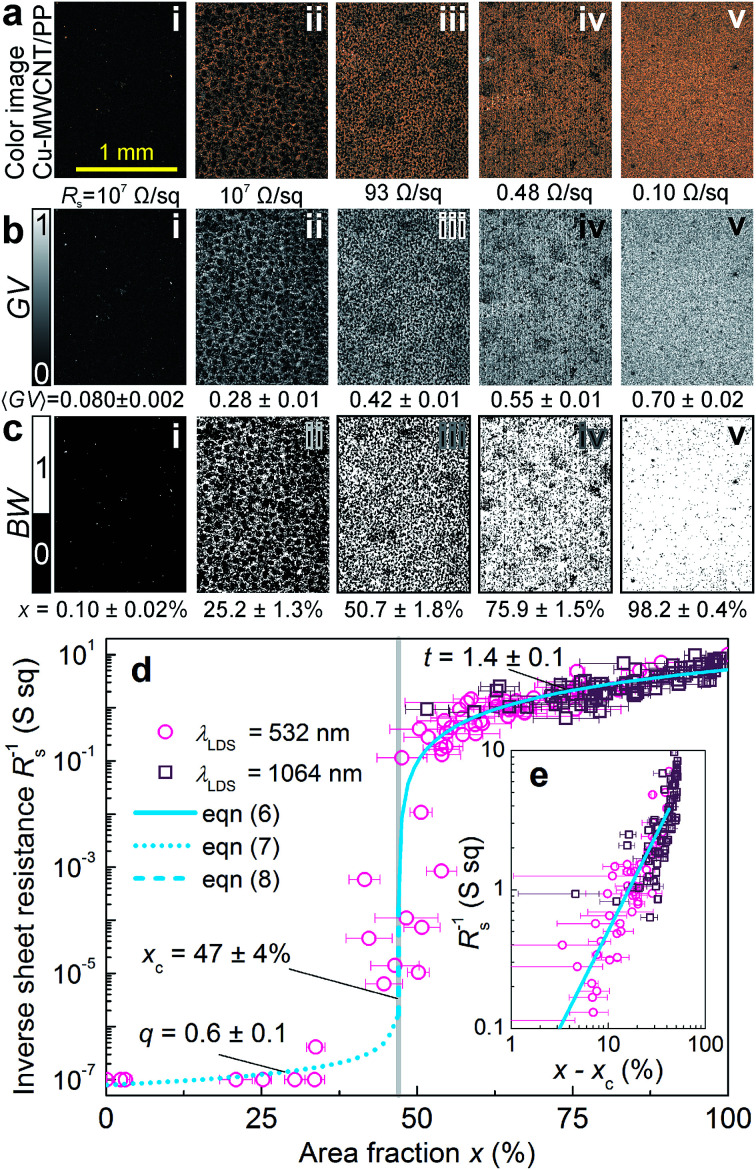
(a) RGB color digital optical microscope images of the Cu layer on MWCNT/PP nanocomposite substrate. The measured sheet resistance values are given below each image. The size of each microscope image is 1.31 × 1.74 mm^2^. (b) The color images converted to grayscale images by using eqn (1). The average grayscale value calculated by eqn (2) is given below each image. (c) The grayscale images converted to black-and-white binary images using eqn (4) with the threshold value of GV_th_ = 0.39 ± 0.02 calculated by eqn (3). The area fraction was calculated from black-and-white images by using eqn (5) given below each image. (d) Inverse sheet resistance *versus* area fraction of Cu layer on MWCNT/PP substrate. The open circles and open squares correspond to LDS processing using laser irradiation wavelengths *λ*_LDS_ = 532 nm and *λ*_LDS_ = 1064, respectively. The solid, dot and dash lines are fits of the experimental data point by eqn (6)–(8). The horizontal error bars indicate the standard deviation in the area fraction measurements taken from five sections of the microscope images. (e) The inverse sheet resistance *versus* difference of area fraction and percolation threshold in log–log representation. Linear fit of experimental data points by eqn (6) is given by straight solid line.

The color RGB digital optical microscope pictures of the plastic specimens after the LDS and subsequent ACD and measured sheet resistances are presented in Fig. 2a. The amount of Cu on MWCNT/PP and sheet resistance depended on the laser irradiation dose in LDS treatment.^14^ The Cu deposition quantity increases and sheet resistance deceases from the 10^7^ Ω sq^−1^ (Fig. 2a-i) to the 0.10 Ω sq^−1^ (Fig. 2a-v) approaching intermediate values (Fig. 2a-ii–iv). The appearance of specimen images depended on the area fraction *x* of the plastic surface plated by the metal. The laser patterned MWCNT/PP nanocomposite substrate after LDS, and subsequent ACD procedures with a minimal amount of Cu had nearly black color (Fig. 2a-i). The almost all covered substrate by Cu deposition produced the Cu color (Fig. 2a-v). Openings in the Cu layer on the plastic surface presented the black color of laser structured nanocomposite substrate and metalized parts color of Cu (Fig. 2a-ii–iv). The color images converted to grayscale images by using eqn (1) are given Fig. 2b. The average values of grayscale images were calculated by using eqn (2) and are given below each grayscale image Fig. 2b. The average grayscale value of Cu deposition increases from the 0.080 ± 0.002 (Fig. 2b-i) to the 0.70 ± 0.02 (Fig. 2b-v) and obtain intermediate values (Fig. 2b-ii–iv). The grayscale images converted to black-and-white binary images using eqn (4) and the threshold value of the value GV_th_ = 0.39 ± 0.02 calculated by eqn (3)Fig. 2c. The area fraction was calculated from black-and-white images by using eqn (5) and given below each image Fig. 2c. The area fraction of Cu deposition increases and from the *x* = 0.10 ± 0.02% (Fig. 2c-i) to the *x* = 98.2 ± 0.4% (Fig. 2c-v) and obtain intermediate values (Fig. 2c-ii–iv). The area fraction *x* values correspond well to the amount of Cu visually absorbed from specimen images (Fig. 2a). Inverse sheet resistance *R*_s_^−1^*versus* area fraction *x* of Cu layer on MWCNT/PP substrate is given in Fig. 2d. The inverse sheet resistance has an almost constant value of 10^−7^ S sq for area fraction vales ranging from 0% to 35%. Then the inverse sheet resistance has rapidly increasing random values in the range form 10^−7^ S sq to 10^−1^ S sq at the area fraction values from 35% to 55%. The increase of inverse sheet resistance from 0.1 S sq to 10 S sq is observed starting from area fraction of 55% to 100%. The jump over several orders of magnitude of inverse sheet resistance observed at the area fraction of ≈47% suggested that percolation effect of electrically conductive Cu layer on the insulating MWCNT/PP nanocomposite substrate was observed. The inverse sheet resistance of thin conductive Cu layer and on MWCNT/PP insulating substrate according to percolation model above the percolation threshold can be expressed as:^26–30,37,38^

6 *R*_s_^−1^ = *hσ*_Cu_(*x* − *x*_c_)^*t*^, for *x* > *x*_c_,

where *h* is the thickness of metal and plastic compound, *σ*_Cu_ = 5.9 × 10^5^ S cm^−1^ is the specific electrical conductivity of Cu,^39,40^*x* is the surface area fraction of plastic surface plated by metal, *x*_c_ is the percolation threshold, *t* is the exponential factor. The inverse sheet resistance below the percolation threshold:^28,37,38^

7 *R*_s_^−1^ = *hσ*_MWCNT/PP_(*x*_c_ − *x*)^−*q*^, for *x* < *x*_c_,

where *σ*_MWCNT/PP_ = 4 × 10^−5^ S cm^−1^ is the specific electrical conductivity of MWCNT/PP nanocomposite substrate at 1.5 wt%,^41^*q* is the exponential factor below the percolation threshold.

The inverse sheet resistance at the percolation threshold can be expressed:^28,37^

8 *R*_s_^−1^ = *hσ*_Cu_^1−*u*^*σ*_MWCNT/PP_^*u*^, for *x* = *x*_c_,

where *u* = *t*/(*t* + *q*). The percolation behavior of the inverse sheet resistance of plastic–metal compound defined by eqn (6)–(8) was applied to explain our experimental data by fits in Fig. 2d. The inverse sheet resistance of Cu deposition on the nanocomposite substrate has the power-law type increase *versus* area fraction. The exponents *t* = 1.4 ± 0.1 and *q* = 0.6 ± 0.1 of the power-law and the percolation threshold *x*_c_ = 47 ± 4% were found from the experimental data point fits by eqn (6) and (7) (Fig. 2d). The increase of inverse sheet resistance with a rising Cu area fraction happened due to the larger amount of plated Cu on the specimen. That causes larger values of inverse sheet resistance which is proportional to Cu conductance. The linear dependence of inverse sheet resistance on the difference of area fraction and percolation threshold in log–log representation is given in Fig. 2e. Experimental data points fitted by solid straight line using eqn (6) prove percolation type dependence above the threshold (Fig. 2e). Similar results of sheet resistance dependence on the color-difference between sample images after LISA and subsequent electroless Cu deposition has been reported in our previous work in ref. 10. This power law behavior can be transformed to the quantity of the Cu deposition on the plastic surface. Therefore, the quality of deposited Cu layer by the ACD method can be assessed by digital image processing of specimen photos.

## 3. Conclusions

To conclude, the MWCNT/PP nanocomposite substrate was patterned by the LDS technique. The Cu layer was selectively deposited on the laser-patterned areas by using the ACD technique. The images of samples were digitally processed, and the area fraction of Cu on MWCNT/PP was evaluated. The inverse sheet resistance of Cu layer had the power-law dependence on the difference of area fraction and the percolation threshold. The percolation theory of inverse sheet resistance of metal–plastic compound has been applied for clarification of experiment results. The experimental data coincide well with the percolation equations. A new digital image processing method is a powerful technique for assessment of the quantity and quality of metal deposition.

## Conflicts of interest

There are no conflicts to declare.

## Supplementary Material
